# Value of thyroid volume as a complementary indicator for initial levothyroxine dosing in congenital hypothyroidism: a retrospective cohort study

**DOI:** 10.3389/fped.2026.1893332

**Published:** 2026-07-10

**Authors:** Yuling Zhang, Qingbiao Liu, Huichang Luo, Baoan Lin, Liuhua Liao

**Affiliations:** 1 The First School of Clinical Medicine, Guangdong Medical University, Zhanjiang Guangdong, China; 2 Newborn Disease Screening Center, Huizhou First Maternity and Child Health Hospital, Huizhou, Guangdong, China; 3 Department of Pediatrics, Dongguan Dalang Hospital, Dongguan Guangdong, China; 4 Department of Pediatrics, Tung-Wah-Hospital Affiliated to Sun Yat-sen University, Dongguan Guangdong, China; 5 Department of Pediatrics, Huizhou Central People's Hospital, Huizhou, Guangdong, China

**Keywords:** congenital hypothyroidism, individualized medicine, levothyroxine, thyroid ultrasound, thyroid volume

## Abstract

**Objective:**

This study aimed to generate hypotheses on the effectiveness and safety of pre-treatment thyroid volume as an adjunctive parameter for guiding initial levothyroxine (L-T4) dosing in children with congenital hypothyroidism (CH).

**Methods:**

This retrospective study included children diagnosed with CH at Huizhou First Maternal and Child Health Hospital (2021–2023). Based on first follow-up ultrasound thyroid volume, patients were divided into reduced (A, *n* = 6), normal (B, *n* = 48), and enlarged (C, *n* = 19) groups. Initial L-T4 doses were 11–13 μg/kg/d (A), 6–9 (B), and 9–11 (C). Outcomes included time to thyroid function normalization, L-T4 dose requirements, physical growth, and Gesell Developmental Quotient (DQ) up to 24 months of age.

**Results:**

At screening and before treatment, both Group A and Group C had significantly higher TSH levels and lower FT4 levels than Group B (all *P* < 0.001). In Group C, thyroid volume showed a significant positive correlation with pre-treatment TSH (*r* = 0.705, *P* < 0.001) and a non-significant negative correlation with FT4 (*r* = −0.440, *P* = 0.060). The median time to TSH normalization was 28 days (IQR 28–29.75) in Group A, 14 days (IQR 14–14) in Group B, and 14 days (IQR 14–28) in Group C; FT4 normalization was achieved at 14 days in all groups. Although there were significant differences in L-T4 dose requirements among the three groups (Group A > Group C > Group B), no significant differences in body weight, length/height, or DQ were observed between any CH group and the healthy control group within 24 months of age (*P* > 0.05).

**Conclusion:**

These findings suggest that children with CH and abnormal thyroid volume may require higher initial L-T4 doses and a longer time to TSH normalization. Based on these observations, we propose the following hypothesis: thyroid volume, as an adjunct to thyroid function tests in guiding initial dosing regimens, holds certain research value in clinical practice. Future prospective, multicenter, and large-sample studies are required to validate this hypothesis.

## 1 Introduction

Congenital hypothyroidism (CH) is one of the most common endocrine disorders in childhood, with a global incidence of approximately 1:2,000 to 1:3,000. The condition is primarily caused by thyroid dysgenesis (including ectopy, hypoplasia, or agenesis) or dyshormonogenesis (1). Thyroid hormones are essential for early brain development and physical growth; delayed treatment can lead to irreversible intellectual impairment.

Currently, the diagnosis of CH relies mainly on serum levels of thyroid-stimulating hormone (TSH) and free thyroxine (FT4). Imaging examinations, particularly thyroid ultrasound, provide crucial etiological clues. Although radionuclide scanning is superior for detecting ectopic thyroid tissue, its use in neonates is limited by radiation exposure and procedural complexity (2). In contrast, thyroid ultrasound is non-invasive, convenient, and accurately assesses the location, morphology, and volume of the thyroid gland; it has gradually become the first-line imaging modality for etiological diagnosis of neonatal CH (3, 4). Thyroid volume remains relatively stable during the first three weeks of life; measurements taken at 2–4 weeks of age avoid early physiological fluctuations and provide guidance for timely treatment (5).

Levothyroxine (L-T4) is the standard treatment for CH. European guidelines recommend an initial dose of 10–15 μg/kg/day, stratified according to biochemical severity (4, 6). However, relying solely on TSH/FT4 levels does not reveal the impact of volume abnormalities on functional reserve: markedly reduced volume often indicates insufficient follicular cell mass, whereas increased volume suggests compensatory hyperplasia due to dyshormonogenesis, though function may still be below normal (7, 8). Previous studies have shown that combining TSH levels with thyroid volume helps distinguish permanent from transient CH (9). Therefore, incorporating thyroid volume into initial dosing decisions may enable more accurate prediction of L-T4 requirements and avoid over- or under-treatment. Nevertheless, no guideline has yet formally integrated thyroid volume into initial dosing algorithms. The primary aim of this hypothesis-generating retrospective study is to explore whether neonatal thyroid volume, measured before treatment, is associated with treatment response (TSH/FT4 normalization time and dose requirements) and short-term outcomes in children with CH, and to generate the following prespecified hypothesis for future validation: In children with CH, an initial L-T4 dosing strategy stratified by thyroid volume (reduced, normal, increased) leads to comparable short-term neurodevelopmental and physical growth outcomes while achieving timely TSH normalization, compared with a uniform high-dose regimen. The current study provides preliminary data to test this hypothesis. In particular, we emphasize the role of thyroid ultrasound in the etiological workup, its potential to inform initial dosing decisions, and its relationship with short-term clinical outcomes.

## 2 Materials and methods

### 2.1 Study design and data source

We conducted a retrospective cohort study of children diagnosed with CH at the Newborn Screening Center of Huizhou First Maternal and Child Health Hospital between January 2021 and December 2023. The regional newborn screening program used a TSH cutoff of 9.0 mIU/L (whole-blood sample) for recall. Diagnosis followed the 2020–2021 consensus guidelines on congenital hypothyroidism (6): a TSH level ≥20 mIU/L with decreased or normal FT4 was diagnostic for congenital hypothyroidism. Confirmatory blood samples were obtained via venipuncture, without strict fasting requirement (neonates were typically fed 1–2 h before sampling). TSH and FT4 were measured using a chemiluminescence immunoassay (Cobas e601, Roche Diagnostics) with upper detection limit of 100 mIU/L for TSH.

Eligible participants were full-term infants with ultrasonographically confirmed orthotopic thyroid gland (i.e., normal cervical location), complete clinical records, and whose legal guardians provided written informed consent for long-term treatment and regular follow-up. Children meeting any of the following criteria were excluded: (1) preterm birth (gestational age <37 weeks); (2) any known chronic disease or syndrome affecting physical or neurodevelopmental outcomes (e.g., congenital heart disease, genetic syndromes); (3) known intellectual disability disorder; or (4) inability to adhere to the follow-up protocol.

**Figure 1:**
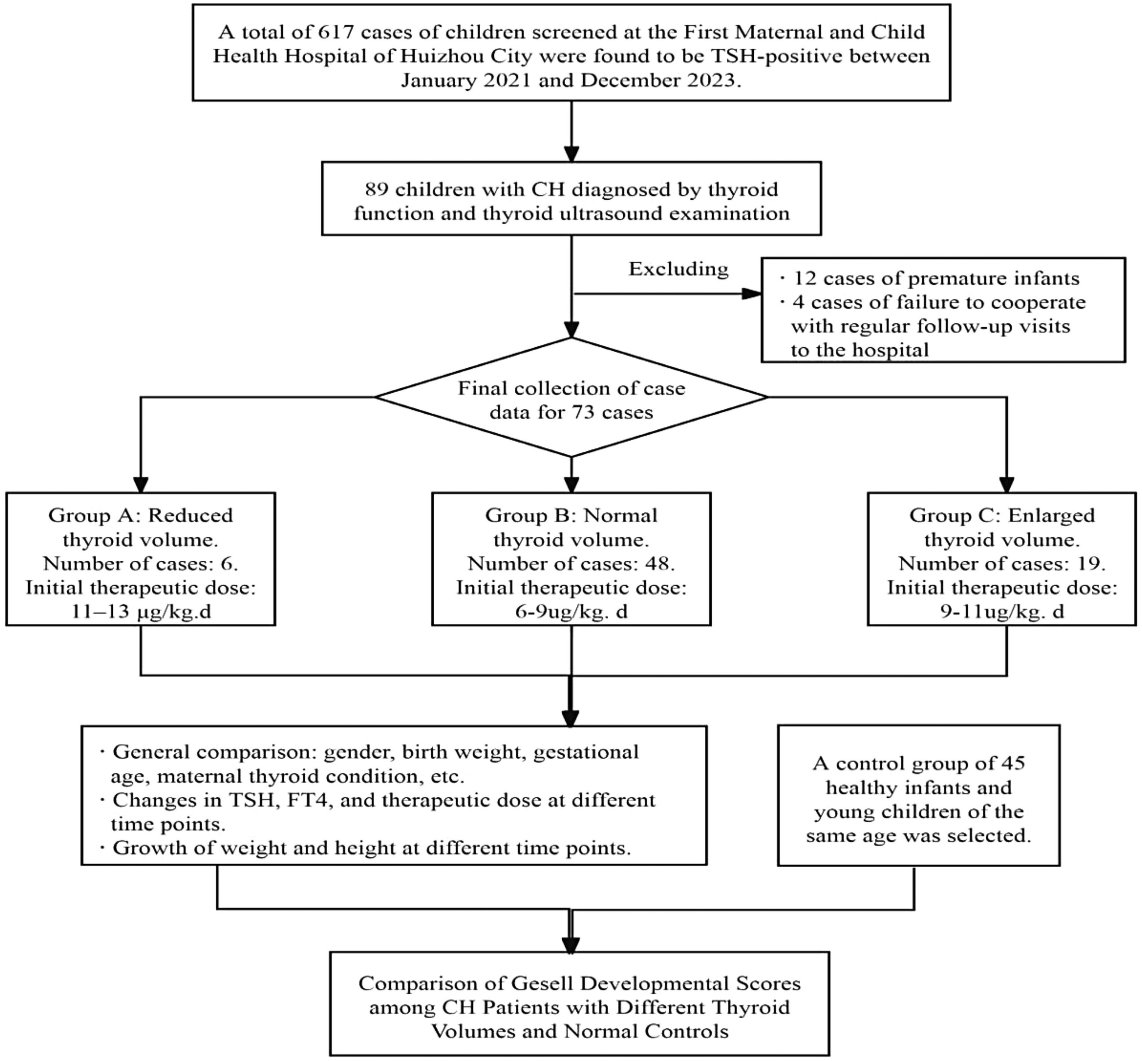
Study design flowchat.volume grouping cutoffs: reduced <0.37 mL; normal 0.37–0.91 mL; enlarged >0.91 mL.

Among 617 neonates with elevated TSH on screening, 89 were diagnosed with CH. After excluding 12 preterm infants and 4 lost to follow-up, 73 children were included. All enrolled patients had an orthotopic thyroid gland (no agenesis or ectopy). Additionally, 45 age- and sex-matched healthy children without thyroid or developmental disorders were concurrently enrolled from the Department of Child Health Care as a reference population for neurodevelopmental evaluation. The study design and participant selection process are illustrated in Figure 1. In the figure, the volume ranges for each group are indicated in parentheses: reduced <0.37 mL, normal 0.37–0.91 mL, enlarged >0.91 mL. This study was approved by the Ethics Committee of Huizhou First Maternal and Child Health Hospital (Approval No. 20250916 (A1)).

### 2.2 Thyroid ultrasound examination and patient grouping

All patients underwent thyroid ultrasonography before treatment initiation (at the first follow-up visit, on postnatal days 14–28; median age 15 days, IQR 12–18 days). Examinations were performed by two experienced attending physicians following a standardized protocol using the same equipment (Mindray DC-8 Pro color Doppler ultrasound system with an L12-3E linear array probe). During the examination, the infant was placed in the supine position. The thyroid gland was scanned at high frequency using the thyroid preset to assess morphology, size, echogenicity, and intraglandular blood flow signals. Three measurements of each lobe's dimensions were taken, and the average value was used. To assess measurement consistency, 20 randomly selected infants underwent repeated measurements; the intraclass correlation coefficient (ICC) for thyroid volume was 0.94 (95% CI: 0.89–0.97).

Thyroid volume was calculated using the ellipsoid formula (10): volume (mL) = length (cm) × width (cm) × thickness (cm) × 0.479/1,000. Based on established reference ranges for thyroid volume in healthy Chinese neonates (mean ± SD: 0.64 ± 0.27 mL) (10), the children were divided into three groups (Table 1). Group A (reduced volume): volume < mean − 1 SD, i.e., <0.37 mL. Actual volume: 0.22 ± 0.06 mL (*n* = 6); all infants in this group had ultrasonographically confirmed hypoplasia (no agenesis or ectopy). Group B (normal volume): volume within mean ±1 SD (0.37–0.91 mL). Actual volume: 0.69 ± 0.06 mL (*n* = 48). Group C (increased volume): volume > mean +1 SD, i.e., >0.91 mL. Actual volume: 2.42 ± 0.71 mL (*n* = 19).

**Table 1:** Thyroid ultrasound examination of three groups of CH children.

Volume grouping	Right lobe			Left lobe			Total volum(mL)
	Length (mm)	Width(mm)	Thickness (mm)	Length (mm)	Width (mm)	Thickness (mm)	
A:reduction (*n* = 6)	8.0 ± 0.89	5.50 ± 1.22	5.17 ± 1.17	7.83 ± 0.75	5.50 ± 0.84	5.17 ± 0.75	0.22 ± 0.06
B:normal (*n* = 48)	16.0 ± 1.11	7.17 ± 0.52	6.42 ± 0.49	15.98 ± 1.12	7.13 ± 0.53	6.31 ± 0.47	0.69 ± 0.06
C:enlargment (*n* = 19)	19.53 ± 1.22	12.32 ± 1.83	10.74 ± 1.79	19.37 ± 1.54	11.84 ± 2.19	10.47 ± 2.01	2.42 ± 0.71

### 2.3 Initial L-T4 administration and follow-up protocol

All children started L-T4 treatment immediately after diagnosis (all within 2–4 weeks of birth). Because this was a retrospective study, the initial L-T4 dose was not assigned prospectively according to thyroid volume. Instead, the treating physicians determined the dose based on clinical judgment, guideline recommendations, and baseline TSH/FT4 levels. For the purpose of this analysis, we retrospectively grouped patients by their first ultrasound volume and compared the actual doses administered, which, as shown in the results, exhibited a pattern consistent with volume-based stratification. The dose ranges described below (11–13, 6–9, 9–11 μg/kg/d) represent the observed ranges in each volume group, not a protocol-driven allocation. Referring to the 2021 European consensus guidelines on congenital hypothyroidism (6), we observed that in practice, the doses given to each group fell into the following ranges: Group A (reduced volume) received a high dose of 11–13 μg/kg/day (likely due to severely insufficient follicular cell mass); Group B (normal volume) received a lower dose of 6–9 μg/kg/day; Group C (increased volume) received an intermediate dose of 9–11 μg/kg/day. Within these ranges, fine-tuning (±1–2 μg/kg/day) was permitted based on baseline FT4 severity according to an internal departmental protocol: increase by 1 μg/kg/day when FT4 < 5.0 pmol/L, and decrease by 1 μg/kg/day when FT4 > 15 pmol/L.

After treatment initiation, thyroid function (TSH, FT4) was rechecked every two weeks until normalization. TSH normalization time was defined as the first time TSH fell below the age-specific upper limit of normal, confirmed by a second measurement two weeks later. FT4 normalization time was defined as the first time FT4 rose above the age-specific lower limit of normal (>11.5 pmol/L), also confirmed by a second measurement two weeks later. Subsequently, follow-up was conducted according to guidelines: every 1–3 months during the first year of life, every 2–4 months during the second year, and every 3–6 months thereafter. L-T4 doses were adjusted to maintain TSH and FT4 within age-specific reference ranges. The TSH assay used in this study was a chemiluminescence method with an upper detection limit of 100 mIU/L; values exceeding this limit were recorded as 100 mIU/L.

### 2.4 Physical and neurodevelopmental assessments

At 1, 3, 6, 12, 18, and 24 months of age, anthropometric measurements (body weight and length/height) were performed using standardized equipment and techniques. Neurocognitive development was assessed at the same time points using the Gesell Developmental Scale by an evaluator who was blinded to the children's group assignments. This scale is applicable to children aged 4 weeks to 6 years and evaluates five domains: gross motor, fine motor, adaptive behavior, language, and personal-social behavior. Overall performance was expressed as the developmental quotient (DQ), with a DQ ≥ 85 defined as normal. A reference population of 45 healthy children underwent the same assessments.

### 2.5 Statistical analysis

SPSS 26.0 software was used for statistical analysis. Normality was tested using the Shapiro–Wilk test. Normally distributed continuous variables are presented as mean ± standard deviation and were compared using one-way analysis of variance (ANOVA) with Bonferroni *post-hoc* correction. Non-normally distributed continuous variables are presented as median with interquartile range [M(Q1, Q3)] and were compared using the Kruskal–Wallis *H*-test with Dunn's *post-hoc* Bonferroni correction. Categorical variables were analyzed using the chi-square test or Fisher's exact test. Spearman's rank correlation analysis was performed to evaluate the relationship between thyroid volume and thyroid function parameters measured at the first follow-up visit within each subgroup.

## 3 Results

### 3.1 Baseline characteristics

No statistically significant differences were observed among the three groups in terms of sex distribution, gestational age, maternal thyroid function status during pregnancy, birth weight, birth length, or age at diagnosis (*P* > 0.05; Table 2). All children started treatment within 2–4 weeks after birth, and there was no significant difference in the age at treatment initiation among the groups (*P* > 0.05).

**Table 2:** General information of three groups of children.

General information	Group A (*n* = 6)	Group B (*n* = 48)	Group C (*n* = 19)	*χ^2^/H/F*	*P*
Gender [*n* (%)]				3.249	0.211^a^
male	2 (33.33)	28 (58.33)	7 (36.84)		
female	4 (66.67)	20 (41.67)	12 (63.16)		
Gestational age [weeks,median (Q1,Q3)]	39.00 (37.75,39.25)	39.00 (38.00,40.00)	39.00 (38.00,40.00)	0.407	0.816^b^
Maternal hyperthyroidism [*n* (%)]	0 (0.00)	2 (4.17)	0 (0.00)	0.949	1.000^a^
Maternal hypothyroidism [*n* (%)]	1 (16.67)	1 (2.08)	1 (5.26)	3.336	0.137^a^
Birth weight (kg, mean ± SD)	2.81 ± 0.41	3.20 ± 0.43	3.18 ± 0.39	2.270	0.111^c^
Birth length [cm, median (Q1,Q3)]	49.00 (46.50,50.50)	50.00 (49.00,52.00)	51.00 (49.00,52.00)	3.003	0.223^b^
Age at diagnosis (days, mean ± SD)	11.33 ± 3.93	12.65 ± 4.72	13.63 ± 3.85	0.685	0.507^c^

a chi-square test or Fisher’s exact test.

b Kruskal Wallis test.

c one-way ANOVA. H is the Kruskal–Wallis test statistic, and F is the F statistic of ANOVA. Group A: reduced thyroid volume, Group B: normal thyroid volume, Group C: enlarged thyroid volume.

### 3.2 Thyroid function parameters and intergroup comparisons

As shown in Table 3, significant differences were observed among the three groups in screening TSH, pre-treatment (first follow-up) TSH and FT4, and time to TSH normalization (all *P* < 0.001). Both Group A (reduced volume) and Group C (increased volume) had significantly higher screening and pre-treatment TSH levels than Group B, and significantly lower pre-treatment FT4 levels than Group B (all *P* < 0.001). Of note, Group C had the lowest pre-treatment FT4 level (median 4.45 pmol/L), even lower than that of Group A (6.89 pmol/L). The median time to TSH normalization was 28 days (IQR 28–29.75 days) in Group A, 14 days (IQR 14–14 days) in Group B, and 14 days (IQR 14–28 days) in Group C. FT4 recovery time to normal was 14 days in all three groups (median, IQR 14–14 for all). Recovery times for TSH and FT4 were calculated according to the actual follow-up schedule (e.g., biweekly assessments after the first dose). Normalization at the first 2-week visit was recorded as 14 days; if not achieved, the next 2-week check was used, with normalization then recorded as 28 days, and so forth.

**Table 3:** Age and TSH and FT4 levels of three groups of children.

Thyroid function parameters	Group A (*n* = 6)	Group B (*n* = 48)	Group C (*n* = 19)	*H*	*P*
Age at start of treatment [days,median (Q1,Q3)]	11.50 (7.75,16.50)	13.00 (9.00,16.75)	15.00 (12.00,17.00)	2.483	0.289
TSH initial screening value [mIU/L,median (Q1,Q3)]	173.50 (16.83,260.00)	12.40 (10.20,17.98)^a^	29.20 (16.20,56.20)^b^	20.279	<0.001
TSH re-examination value [mIU/L, median (Q1,Q3)]	100.00 (79.54,100.00)	29.90 (24.30,45.39)^a^	100.00 (77.46,100.00)^b^	38.975	<0.001
TSH recovery time to normal [days, median (Q1,Q3)]	28.00 (28.00,29.75)	14.00 (14.00,14.00)^a^	14.00 (14.00,28.00)^a^	18.641	<0.001
FT4 re-examination value [pmol/L, median (Q1,Q3)]	6.89 (2.71,11.96)	15.26 (11.63,19.08)^a^	4.45 (3.07,10.58)^b^	28.493	<0.001
FT4 recovery time to normal [days, median (Q1,Q3)]	14.00 (14.00,14.00)	14.00 (14.00,14.00)	14.00 (14.00,14.00)	0.00	1.00

a Compared to the group A *P* < 0.05.

b Compared to the group B *P* < 0.05, Kruskal Wallis test was used for inter group comparisons, and Bonferroni correction was used for multiple comparisons. H is the Kruskal–Wallis test statistic. The “TSH/FT4 re-examination values” refer to the detection values at the first follow-up (before treatment). In this study, the upper limit of TSH detection was 100 mIU/L, and values exceeding this range were recorded as 100. Therefore, the median TSH values in the volume reduction group and the enlargement group appear as 100. Group A: reduced thyroid volume, Group B: normal thyroid volume, Group C: enlarged thyroid volume.

### 3.3 Correlation between thyroid volume and first follow-up thyroid function parameters

To explore the relationship between thyroid volume and thyroid function beyond intergroup differences, Spearman correlation analyses were performed (Table 4). In Group C (increased thyroid volume), thyroid volume showed a significant positive correlation with the first follow-up TSH value (*r* = 0.705, *P* < 0.001) and a negative correlation with FT4 that did not reach statistical significance (*r* = −0.440, *P* = 0.060). In Group B (normal thyroid volume), no significant correlations were observed between thyroid volume and first follow-up TSH (*r* = 0.117, *P* = 0.430) or FT4 (*r* = −0.192, *P* = 0.190). In Group A (reduced thyroid volume), no significant correlations were found with TSH (*r* = −0.686, *P* = 0.132) or FT4 (*r* = 0.406, *P* = 0.425). These findings indicate that a significant association between increased thyroid volume and elevated TSH level was present only in the group with thyroid enlargement.

**Table 4:** Correlation analysis of thyroid volume with first follow-up TSH and FT4.

Indicator	Symbol	Group A		Group B		Group C	
		TSH	FT4	TSH	FT4	TSH	FT4
Correlation coefficient	*r*	−0.686	0.406	0.117	−0.192	0.705**	−0.440
Significance	*p*	0.132	0.425	0.430	0.190	<0.001	0.060
LCL		−0.964	−0.626	−0.182	−0.459	0.356	−0.751
UCL		0.314	0.921	0.395	0.106	0.881	0.033
cases	*n*	6	6	48	48	19	19

The correlation coefficient with volume was calculated using Spearman's rank correlation analysis. LCL is 95% CI lower bound;UCL is 95% CI upper bound.

### 3.4 Changes in TSH levels

**Figure 2:**
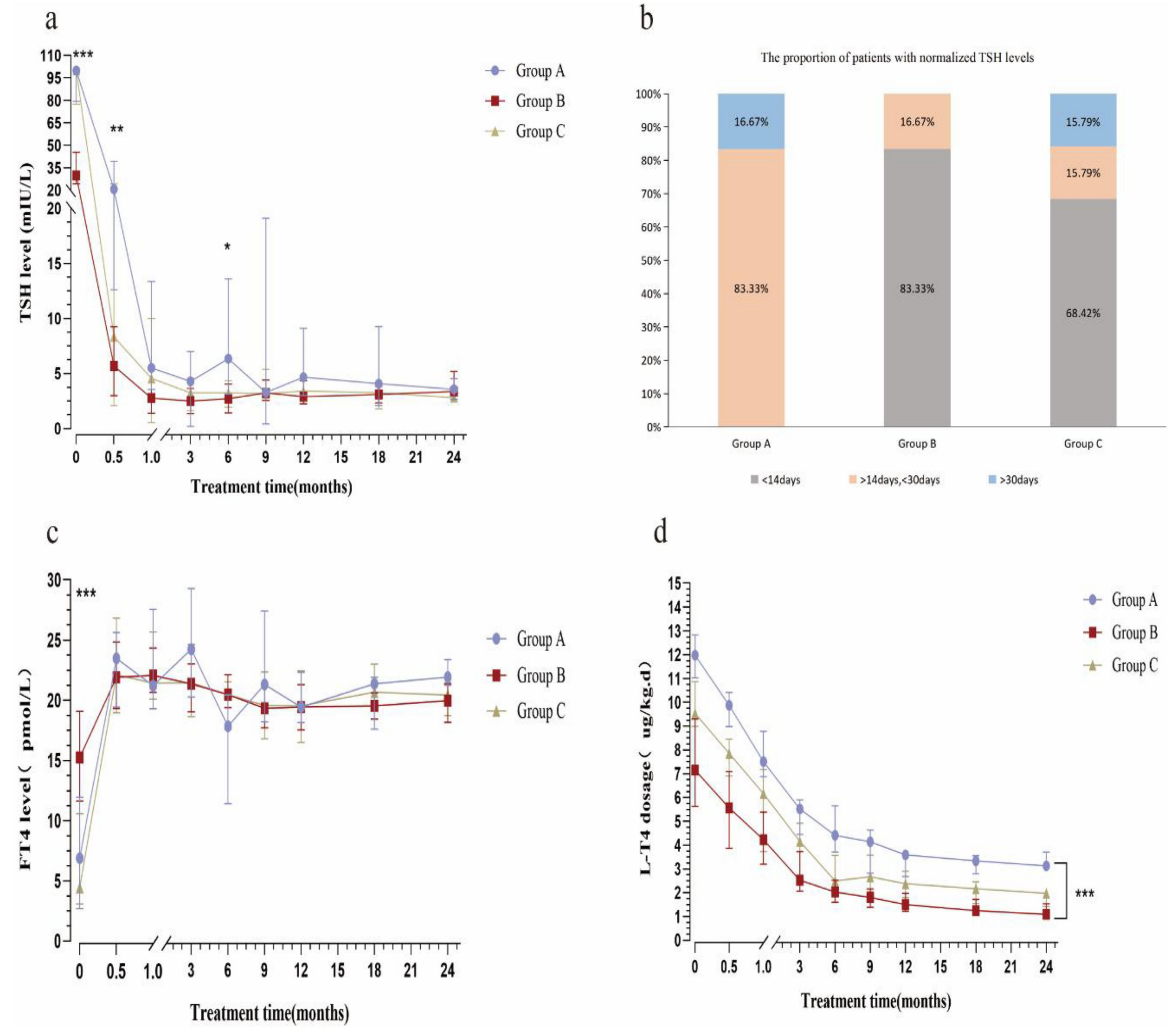
**(a–d)** TSH and FT4 levels and treatment recovery of patients. **(a)** Changes in TSH values during treatment. **(b)** The proportion of patients with normalized TSH levels. **(c)** Changes in FT4 values during treatment. **(d)** Changes in L-T4 dosage during treatment.Group A: reduced thyroid volume, Group B: normal thyroid volume, Group C: enlarged thyroid volume. Asterisk indicate statistically significant differences in defferents at the level of 0.05, * represents *p* < 0.05, ** represents *p* < 0.01, *** represents *p* < 0.001.

Significant differences in TSH levels were observed among the three groups before treatment and two weeks after treatment initiation (*P* < 0.05). Before treatment, TSH concentrations in Groups A and C were higher than that in Group B. Two weeks after treatment, TSH levels in Group A (reduced volume) decreased but remained above the normal range, whereas levels in Group B (normal volume) and Group C (increased volume) had normalized. At six months of treatment, TSH levels in Group A again exceeded those in the other two groups. No significant intergroup differences were detected at 1, 3, 9, 12, 18, or 24 months of follow-up (*P* > 0.05). These patterns are illustrated in Figure 2a. Regarding the proportion of patients achieving TSH normalization: 83.33% of Group A normalized within 14–30 days, 83.33% of Group B normalized within 14 days, and 68.42% of Group C normalized within 14 days, as detailed in Figure 2b.

### 3.5 Changes in FT4 Levels

Before treatment, the median FT4 value was 4.45 pmol/L in Group C and 6.89 pmol/L in Group A, both well below the normal reference range (for infants ≤4 months of age: 11.5–28.3 pmol/L), whereas the value in Group B was within the normal range. Two weeks after treatment initiation, FT4 levels returned to normal in all groups. At six months of treatment, FT4 levels in the reduced-volume group (Group A) showed a tendency to fluctuate but remained within the normal range (for infants aged 4–12 months: 12.3–22.8 pmol/L). No statistically significant differences were observed among the groups at any time point (*P* > 0.05). These trends are presented in Figure 2c.

### 3.6 L-T4 dose adjustments

Significant differences in L-T4 dosage were observed among the three groups at treatment initiation and at all follow-up time points up to 24 months (*P* < 0.05). Throughout the observation period, the dose requirements consistently followed the pattern of Group A (reduced volume) > Group C (increased volume) > Group B (normal volume), as shown in Figure 2d.

### 3.7 Physical growth

**Figure 3:**
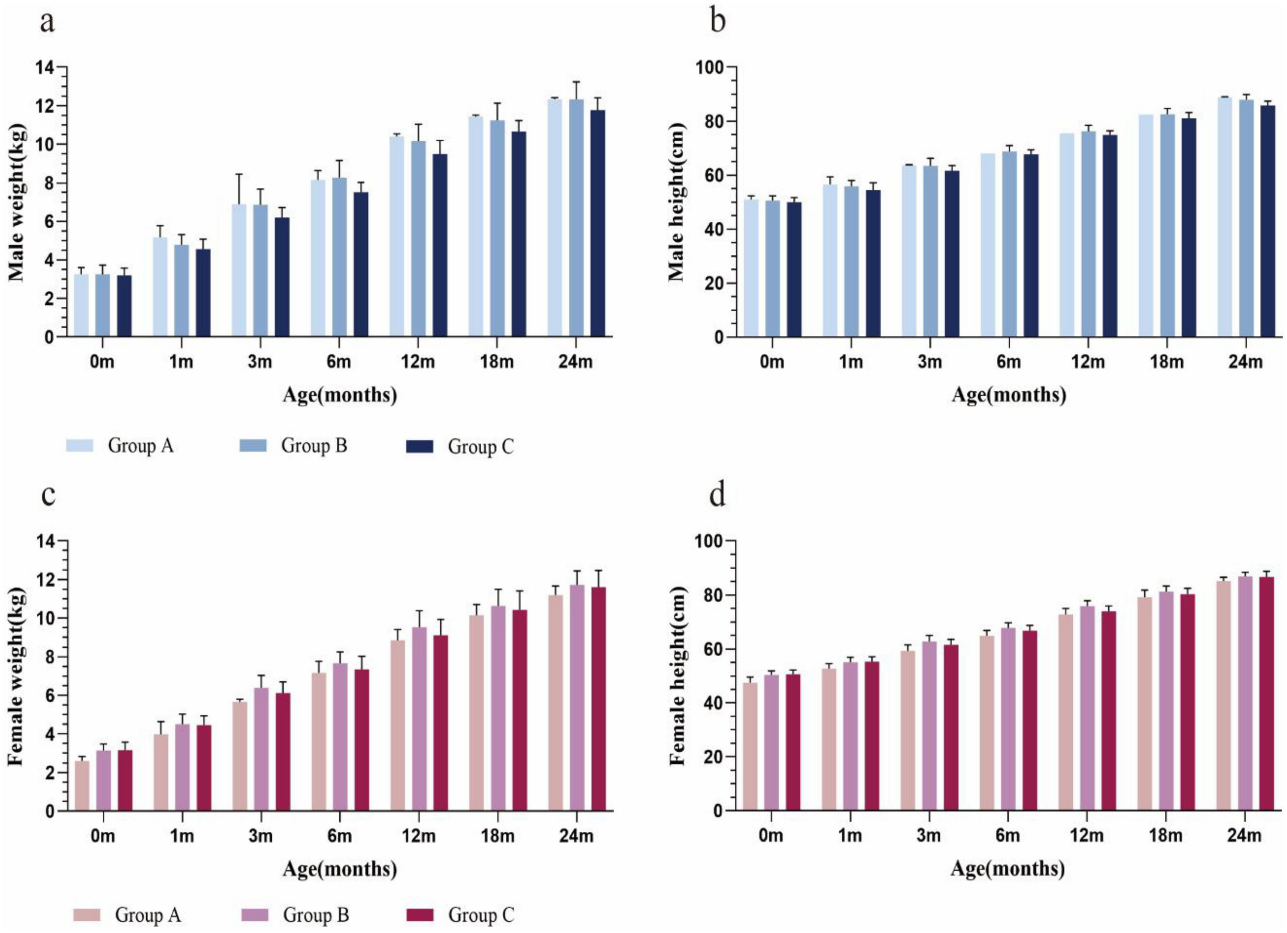
**(a–d)** weight and length/height trajectories of three groups of children compared with WHO growth standards (2006). **(a)** Male weight, **(b)** Male length/height, **(c)** Female weight, **(d)** Female length/height. Group A: reduced thyroid volume, Group B: normal thyroid volume, Group C: enlarged thyroid volume.

At any assessment time point, no statistically significant differences in body weight or length/height were detected between males and females before or after treatment, nor among the three thyroid volume groups (*P* > 0.05). The growth trajectories are shown in Figure 3.

### 3.8 Neurodevelopmental outcomes

Neurodevelopmental assessments performed using the Gesell Developmental Scale at 1, 3, 6, 12, 18, and 24 months of age showed that the developmental quotient (DQ) values in all three groups were within the normal range (DQ ≥ 85). No significant differences were observed when compared with the age-matched healthy control group (*P* > 0.05). These results are summarized in Table 5.

**Table 5:** Gesell developmental quotient(DQ) at diferent ages for the patients and control group.

DQ	Group A (*n* = 6)	Group B (*n* = 48)	Group C (*n* = 19)	Control group (*n* = 45)	*F*	*P*
1 m	88.83 ± 4.49	87.79 ± 3.99	86.16 ± 5.26	87.60 ± 4.14	0.893	0.447
3 m	88.33 ± 3.83	85.58 ± 5.64	86.16 ± 4.73	85.18 ± 4.83	0.738	0.531
6 m	92.67 ± 3.14	91.15 ± 4.44	92.26 ± 3.09	91.22 ± 3.92	0.586	0.625
12 m	93.00 ± 1.26	92.29 ± 4.30	91.05 ± 4.13	92.80 ± 4.25	0.839	0.475
18 m	90.33 ± 2.50	92.35 ± 3.26	92.05 ± 2.68	92.11 ± 3.00	0.785	0.504
24 m	92.33 ± 2.58	92.69 ± 2.83	90.79 ± 3.66	91.62 ± 2.36	2.436	0.168

Groups were compared using one-way ANOVA. Group A: reduced thyroid volume, Group B: normal thyroid volume, Group C: enlarged thyroid volume. Control group: healthy infants.

## 4 Discussion

Early diagnosis of congenital hypothyroidism (CH) relies on the integration of biochemical testing and imaging. Color Doppler ultrasound is widely used due to its non-invasiveness, lack of radiation, and ease of operation (11). Although radionuclide scanning is superior for detecting ectopic thyroid tissue, ultrasound provides a more reliable assessment of thyroid volume and morphological characteristics (12). Therefore, performing thyroid ultrasound at birth or during the neonatal period helps elucidate the etiology of CH, particularly in distinguishing thyroid dysgenesis from *in situ* developmental abnormalities such as dyshormonogenesis (3, 13, 14). The main findings of this study are as follows: (1) The degree of deviation of thyroid volume from the normal range was associated with screening and first follow-up TSH and FT4 levels; (2) Only in the group with increased thyroid volume was there a significant positive correlation between thyroid volume and first follow-up TSH (Spearman *r* = 0.705, *P* < 0.001), and a negative correlation with FT4 that did not reach statistical significance (*r* = −0.440, *P* = 0.060), which generates the hypothesis that volume enlargement does not necessarily indicate adequate functional compensation; (3) The reduced-volume group required a higher starting dose (11–13 μg/kg·d) and a longer time to TSH normalization (28 days), whereas the normal-volume and increased-volume groups required only 14 days; (4) Short-term physical growth and neurodevelopmental outcomes appeared favorable in all three groups, with no significant differences compared with healthy controls.

### 4.1 Thyroid volume as a structural indicator of functional reserve

In this study, both the reduced-volume group (Group A) and the increased-volume group (Group C) had significantly higher TSH levels at initial screening and first follow-up, as well as significantly lower FT4 levels, compared with the normal-volume group (Group B), suggesting that both types of structural abnormalities lead to varying degrees of functional impairment. From a pathophysiological perspective, mutations in genes such as *DUOX2*, *TPO*, and *TSHR* can cause thyroid developmental defects and morphological changes, resulting in partial or complete loss of secretory function (7). In thyroid hypoplasia, the total number of follicular cells is congenitally insufficient, leading to FT4 levels far below the body's basic requirements. This relieves the negative feedback inhibition on the pituitary and hypothalamus, causing an extreme elevation of TSH. However, compensatory mechanisms are largely ineffective, resulting in a classic decompensated state characterized by markedly elevated TSH and significantly reduced FT4 (15). In contrast, the mechanism underlying thyroid enlargement is predominantly dyshormonogenesis: due to a congenital defect in an enzyme involved in iodine transport or thyroid hormone synthesis, the thyroid cannot produce adequate amounts of hormones (16). The consequent reduction in FT4 is sensed by the pituitary, which secretes large amounts of TSH. Prolonged high TSH stimulation leads to follicular cell hyperplasia and hypertrophy, forming a goiter (17). However, our correlation analysis revealed that within Group C, larger volume was paradoxically associated with higher TSH (*r* = 0.705, *P* < 0.001) and a trend toward lower FT4 (*r* = −0.440, *P* = 0.060). This indicates that the hyperplastic response is functionally insufficient; the enlarged gland still fails to produce adequate hormone, and residual function is not simply proportional to size. Notably, in Group A we did not find a significant correlation between volume and TSH (*r* = −0.686, *P* = 0.132), likely due to the small sample size and TSH assay ceiling (100 mIU/L) that truncated extreme values. These findings underscore that thyroid volume reflects structural capacity, but its relationship with actual secretory function is complex and influenced by etiology, duration of TSH stimulation, and individual enzymatic defects. Therefore, volume should be interpreted as a supplementary piece of information, not as a substitute for direct functional measurements.

### 4.2 Clinical implications of volume-stratified dosing in the context of existing evidence

Early initiation of L-T4 therapy (within the first month of life) helps reduce the risk of intellectual impairment in children with CH (18). Current guidelines recommend an initial dose of 10–15 μg/kg·d to achieve rapid TSH normalization (6). However, a uniform high-dose regimen may induce iatrogenic hyperthyroidism in approximately 36.5% of patients, leading to frequent dose adjustments and potentially prolonging TSH recovery time (19). Overtreatment has been associated with behavioral problems at school age (e.g., impulsivity, attention-deficit/hyperactivity disorder), whereas undertreatment may increase the risk of neurodevelopmental disorders such as autism spectrum disorder (20). Consequently, an increasing number of researchers advocate for individualized dosing strategies based on baseline biochemical severity (21, 22). Günbey et al. reported that patients with severely decreased FT4 required higher starting doses and longer TSH recovery times (approximately 30 days) (23), Li et al. also confirmed the feasibility of a stratified dosing protocol based on FT4 levels (24).

Our findings extend this framework by incorporating an anatomical dimension. We observed that infants in the reduced thyroid volume group (Group A) required the highest doses (11–13 μg/kg/day) and took up to 28 days to achieve TSH target levels—a profile similar to that of the biochemically most severe patients in previous studies. In contrast, those in the normal-volume group (Group B) could be safely started on lower initial doses (6–9 μg/kg/day) without prolonging the time to target (14 days). The enlarged-volume group (Group C) required intermediate doses (9–11 μg/kg/day), with most infants reaching TSH target within 14 days. These patterns suggest that incorporating volume assessment may refine initial dose selection, particularly in identifying patients with normal gland size who might be at risk of overtreatment under a fixed high-dose regimen. For infants with extremely low FT4 (e.g., <5 pmol/L), the upper end of the recommended range (13–15 μg/kg/day) should still be used, regardless of thyroid volume.

It must be emphasized that thyroid volume can assist in assessing disease severity but should never replace functional assessment by TSH and FT4. As shown in Table 3, FT4 levels varied widely within the same volume group (e.g., the interquartile range of Group B was 11.63–19.08 pmol/L), indicating that children with the same thyroid volume may exhibit markedly different functional status due to differences in residual function, receptor sensitivity, or hormone synthesis efficiency. Our strategy has always been to interpret volume together with baseline TSH and FT4, not to substitute volume for functional indicators. Future research may further integrate thyroid ultrasound, genetic testing, and thyroid function parameters to guide individualized treatment more precisely.

### 4.3 Comparisons with studies on transient and permanent CH

Previous studies have shown that thyroid volume may help differentiate transient from permanent congenital hypothyroidism (CH). Ackah et al. reported that a thyroid volume ≤0.36 mL or ≥2.5 mL, combined with a diagnostic TSH ≥200 mIU/L, yielded a sensitivity of 78.4% and a specificity of 85.7% for predicting permanence (9). Our grouping cutoffs (reduced <0.37 mL, enlarged >0.91 mL) are broadly consistent with these thresholds. Although we did not perform a levothyroxine withdrawal test (follow-up was limited to 24 months), we observed that Group A consistently required higher doses throughout the study period, which aligns with the view that reduced volume suggests permanent hypoplasia. In contrast, some infants in the enlarged-volume group may have transient dyshormonogenesis; however, they still needed higher doses than those in the normal-volume group during the initial months, likely because the synthetic defect imposes a functional bottleneck. These observations support the value of early ultrasonography in providing prognostic clues before a definitive diagnosis of permanence can be established.

### 4.4 Limitations and acknowledgment of non-significant correlations

Although the three groups differed in TSH normalization time and dose requirements, no physical or neurodevelopmental impairments were observed in any subgroup up to 24 months of age. This favorable outcome may be attributable to early intervention within the first month of life. Nevertheless, this study has several limitations. First, this was a single-center retrospective study with a small sample size in the reduced-volume group (Group A), which may have led to insufficient statistical power, particularly in subgroup correlation analyses (e.g., the correlation coefficient between TSH and volume in Group A was −0.686, yet *P* = 0.132), raising the possibility of a type II error (false negative). More importantly, the lack of statistically significant correlations between thyroid volume and baseline TSH/FT4 in Groups A and B, and the only significant correlation with TSH (but not FT4) in Group C, limits the generalizability of volume as a standalone predictor. This finding suggests that the relationship between volume and function is complex and likely influenced by multiple factors (e.g., etiology, residual function, iodine status). Therefore, thyroid volume should be considered as an adjunctive, not primary, parameter in clinical decision-making.

Second, the chemiluminescence assay used in this study had an upper detection limit of 100 mIU/L; values exceeding this limit were recorded as 100 mIU/L, resulting in censoring of pre-treatment TSH values in Group A and some patients in Group C. This may partly explain why no significant correlation between TSH and volume was observed in Group A (*r* = −0.686, *P* = 0.132); a broader-range assay might have revealed a stronger correlation. The 95% confidence interval for this correlation coefficient ranged from −0.964 to 0.314, indicating substantial instability. Therefore, conclusions regarding Group A should be interpreted with caution, and prospective, multicenter, large-sample studies are needed for confirmation.

Third, individual urinary iodine concentrations were not measured. However, all patients came from Huizhou City, Guangdong Province, China, a region that has long implemented universal salt iodization; multiple monitoring data have shown a median urinary iodine concentration >100 μg/L among school-aged children, indicating an iodine-sufficient area (25). Thus, iodine deficiency is unlikely to have significantly affected volume measurements.

Fourth, the initial doses were not prospectively assigned by volume; they reflected real-world practice, which might have been influenced by the clinicians' awareness of ultrasound findings. Although we observed a dose pattern consistent with volume groups, this does not prove that volume-guided dosing is superior. Finally, the follow-up period (24 months) is too short to assess final intellectual outcomes or to differentiate permanent from transient CH definitively. Longer-term studies with neurocognitive assessments at school age are warranted.

## 5 Conclusion

This hypothesis-generating study suggests that thyroid volume assessment in neonates with CH may provide complementary information for initial L-T4 dosing decisions. Based on our observations, we propose the following hypothesis for future confirmatory research: combining thyroid volume with baseline TSH and FT4 in initial dose selection might help reduce the risk of treatment-related adverse effects and enable individualized management. It must be emphasized that thyroid volume should always serve as an adjunct to, rather than a replacement for, FT4/TSH measurements; for severely affected patients with extremely low FT4 levels, biochemical severity remains the primary determinant. We recommend that neonatal thyroid ultrasound be incorporated into the initial evaluation workflow for CH only within the context of prospective studies designed to test the above hypothesis.

## Data Availability

The raw data supporting the conclusions of this article will be made available by the authors, without undue reservation.
